# Analgesic, Anti-Inflammatory and Anticancer Activities of Extra Virgin Olive Oil

**DOI:** 10.1155/2013/129736

**Published:** 2013-12-23

**Authors:** Myriam Fezai, Laura Senovilla, Mohamed Jemaà, Mossadok Ben-Attia

**Affiliations:** ^1^Laboratoire de Biosurveillance de l'Environnement (LR01/ES14), Equipe REMP “Risque Écologique des Médicaments & Pesticides”, Faculté des Sciences de Bizerte, Université de Carthage, 7021 Zarzouna, Tunisia; ^2^INSERM, U848, 94805 Villejuif, France; ^3^Institut Gustave Roussy, 94805 Villejuif, France; ^4^CNRS UMR5237, CRBM 1919 Route de Mende, 34293 Montpellier, France

## Abstract

*Background*. In folk medicine, extra virgin olive oil (EVOO) is used as a remedy for a variety of diseases. This study investigates the *in vivo* antinociceptive, anti-inflammatory, and anti-cancer effects of EVOO on mice and rats. *Materials and Methods*. In this experimental study, using the acetic acid-induced writhing and formalin tests in mice, the analgesic effect of EVOO was evaluated. Acetylsalicylic acid and morphine were used as standard drugs, respectively. The anti-inflammatory activity was investigated by means of the carrageenan-induced paw edema model in rats using acetylsalicylic acid and dexamethasone as standard drugs. Last, the xenograft model in athymic mice was used to evaluate the anticancer effect *in vivo*. *Results*. EVOO significantly decreased acetic acid-induced abdominal writhes and reduces acute and inflammatory pain in the two phases of the formalin test. It has also a better effect than Dexamethasone in the anti-inflammatory test. Finally, the intraperitoneal administration of EVOO affects the growth of HCT 116 tumours xenografted in athymic mice. *Conclusion*. EVOO has a significant analgesic, anti-inflammatory, and anticancer properties. However, further detailed studies are required to determine the active component responsible for these effects and mechanism pathway.

## 1. Introduction

The Mediterranean diet rich in nuts, fruits, vegetables, legumes, whole-wheat bread, fish, and olive oil [1] has a well-established beneficial role in health promotion [2]. Virgin olive oil is an integral ingredient of this traditional diet and for centuries Mediterranean people have appreciated his nutritional, medical, and cosmetics benefits [3]. Indeed, virgin olive oil has been used as a folk remedy for combating diseases due to its pharmacological properties including cardioprotective, hypotensive, antihyperglycemic, antimicrobial, and anti-inflammatory effects [4–7]. Nowadays, epidemiological studies on virgin olive oil consumers reveal an important decrease in chronic disease, that is, cardiovascular diseases (CVD) [8], atherosclerosis [9], and some types of cancers especially colorectal and breast cancer [10]. In addition, Mediterranean people show a higher life expectancy compared with olive oil consume less populations worldwide [11]. Virgin olive oil is the oil obtained from the fruit of the olive tree solely by mechanical or physical means (the cold-pressed technique), which do not lead to alterations of the oil. Based on the acidity, the International Olive Oil Council has defined the classification of virgin olive oil as follows: extra virgin olive oil, virgin olive oil, and ordinary virgin olive oil. Thus, oil retains the compounds that the fruit develops in response to environmental stress and especially phenolic compounds [12].

In this study, we decided to focalize on the extra virgin olive oil (EVOO) and to investigate its antinociceptive, anti-inflammatory, and anticancer effects using *in vivo* experimental models, namely, the acetic acid-induced abdominal writhing test as well as formalin test to assess the analgesic activity, the carrageenan-induced hindpaw edema model for the anti-inflammatory activity, and human colon carcinoma cells xenograft in nude mice to evaluate the anticancer effect.

## 2. Materials and Methods 

### 2.1. Chemicals

Extra virgin olive oil (Carapelli, Extra Virgin Olive Oil, Florence, Italy) was purchased from local market. PBS was purchased from Gibco-Invitrogen (Carlsbad, CA, USA). Formalin (formaldehyde stock solution 4% (w/v)) was purchased from Sigma-Aldrich (St Louis, MO, USA) while carrageenin (fresh-prepared) and acetic acid (stock solution at 60.05 mM) were provided from Sigma-Aldrich (Taufkirchen, Germany). Dexamethasone sodium phosphate (0.4% injectable solution) had been supplied from Medis laboratories (Nabeul, Tunisia), Aspirin Acetylsalicylic Acid (10% (w/v) injectable-solution) from (LABESFAL, Laboratorios Almiro, Campo de Besteiros, Portugal) and Morphine Chlorhydrate (1% injectable-solution) purchased from Renaudin Laboratories (Itxassou, France). All the drugs were dissolved in saline. Standard solution imbibante BBC97 (Chimifoto Ornano, Italy) was used for plethysmometer (model 7150, Ugo Basile, Italy).

### 2.2. Cell Lines

Wild type human colon carcinoma HCT 116 cells (kindly provided by Bert Vogelstein, The Ludwig Center for Cancer Genetics and Therapeutics and The Howard Hughes Medical Institute, Johns Hopkins Kimmel Cancer Center, Baltimore, MD, USA) were maintained in McCoy's 5A medium supplemented with 10% fetal calf serum (FCS), 10 mM HEPES buffer, 100 units/mL penicillin G sodium, and 100 *μ*g/mL streptomycin sulfate.

### 2.3. Animals

Albino Wistar female rats weighting (200 g ± 20 g) and albino Swiss male mice with 25 g and 30 g weight ranges, provided by the Pasteur Institute of Tunis, Tunisia, were used for the anti-inflammatory and analgesic activities in this study. Mice and rats were housed in laboratory transparent-plastic cages EBECO (groups of six/cage each). Athymic *nu/nu *female mice (20–25 g), provided by the Institut Gustave Roussy, Villejuif, France, in-house animal facility were used for the xenograft model throughout this study. Mice were kept in Makrolons type III wire mesh laboratory cages (Charles River, Boston, MA, USA). Animals were manipulated in strict compliance with widely accepted ethical guidelines for animal experimentation and kept under poor germ conditions at 24°C and 50–60% humidity and were allowed for food and water *ad libitum *prior to the experiments. Light cycle was artificially controlled to provide 14 h of light (from 06:30 a.m. to 08:30 p.m.).

### 2.4. Analgesic Activity

#### 2.4.1. Acetic-Acid Writhing Test

The abdominal writhing response in mice to intraperitoneal injection of acetic acid was used as one of the well-established basic tests for analgesic activity [13]. Extra virgin olive oil was injected at 8 mL/kg and Aspirin (acetylsalicylic acid) at 3.2 g/kg was administered intraperitoneally (i.p.) 30 min prior to the acetic acid i.p. injection of 1% (v/v) at a dose of 10 mL/kg body weight. Acetylsalicylic acid (ASA) served as a positive control while the negative control animals received a similar volume of saline solution 0.9%. All injection volumes are equal to 200 *μ*L. Groups of 8 mice were placed in transparent boxes during the test and counting of abdominal constrictions started immediately after injection of acetic acid for 30 min. The mice with decreased number of writhes were considered protected. Writhing count permitted the percentage of antinociceptive activity to be expressed according to the following ratio: percent inhibition = (1 − *W*_*t*_/*W*_*c*_) × 100, where *W*_*t*_ and *W*_*c*_ represent the number of writhing movements, measured between the treated and control groups.

#### 2.4.2. Formalin Test in Mice

The formalin-induced nociception test was performed as described previously by Hunskaar and Hole [14] and recently by Nakamoto et al. [15]. Mice were divided into groups of 5 and were injected with 20 *μ*L of 10% formalin into the subplantar space of the left hind paw 30 min after olive oil pretreatment at crescent doses of 0.33 mL/kg, 0.66 mL/kg, 1 mL/kg, and 1.33 mL/kg corresponding, respectively, to 10 *μ*L, 20 *μ*L, 30 *μ*L, and 40 *μ*L. Morphine at a dose of 10 mg/kg was served as a positive control. Mice were observed through glass cages one by one and the time spent in liking, biting, and shaking behaviors was measured in seconds during the early phase (0–5 min) and late phase (5–30 min).

### 2.5. Anti-Inflammatory Activity

#### 2.5.1. Carrageenin-Induced Rat Paw Edema

The anti-inflammatory activity was evaluated using *in vivo* carrageenin-induced rat paw edema model considered as the most conventional test for acute inflammation [16]. Wistar rats were divided into 6 groups, 6 rats each, and were injected into the subplantar space of the left hind paw with 200 mg/kg of ASA and 2 mg/kg of dexamethasone, as positive controls, and normal saline for negative one. Each rat received a subplantar injection of 100 *μ*L. Extra virgin olive oil was administered at decreasing volumes of 100 *μ*L, 50 *μ*L and 25 *μ*L corresponding to 0.5 mL/kg, 0.25 mL/kg, and 0.125 mL/kg. Edema was induced on the left hind paw of the rat by subplantar injection of 100 *μ*L of a solution of 0.6% (w/v) carrageenan. Paw thickness was measured before and after carrageenan injection during 5 hours, using a plethysmometer. Percentile edema inhibition was calculated according to the following formula: percentile inhibition = (1 − *V*_*t*_/*V*_*c*_) × 100, where *V*_*t*_ and *V*_*c*_ represent the mean difference in paw measurement between the treated and control groups.

#### 2.5.2. *In Vivo* Xenograft Models

Athymic *nu/nu* six-week-old female mice (IGR animal facility) were inoculated s.c. in 200 *μ*L of PBS with 2 × 10^6^ HCT116 cells into the lower flank [17]. As soon as tumors reached 125 mm^3^, mice received i.p. 200 *μ*L of PBS 1X or extra virgin olive oil (8 mL/kg) three times a week during 4 weeks. Tumor growth was evaluated twice a week using a caliper. The mean of the tumor volume at each point was normalized in each group to the mean volume measured at the first injection. All animals were maintained in specific pathogen-free conditions and all experiments followed the FELASA guidelines.

#### 2.5.3. Statistical Analysis

All data are expressed as the mean ± SD. The statistical significance of differences between controls and test values was assessed with Student's *t*-test (two tailed). Differences were considered significant if *P* < 0.05.

## 3. Results

### 3.1. Analgesic Activity of Extra Virgin Olive Oil

**Figure 1:**
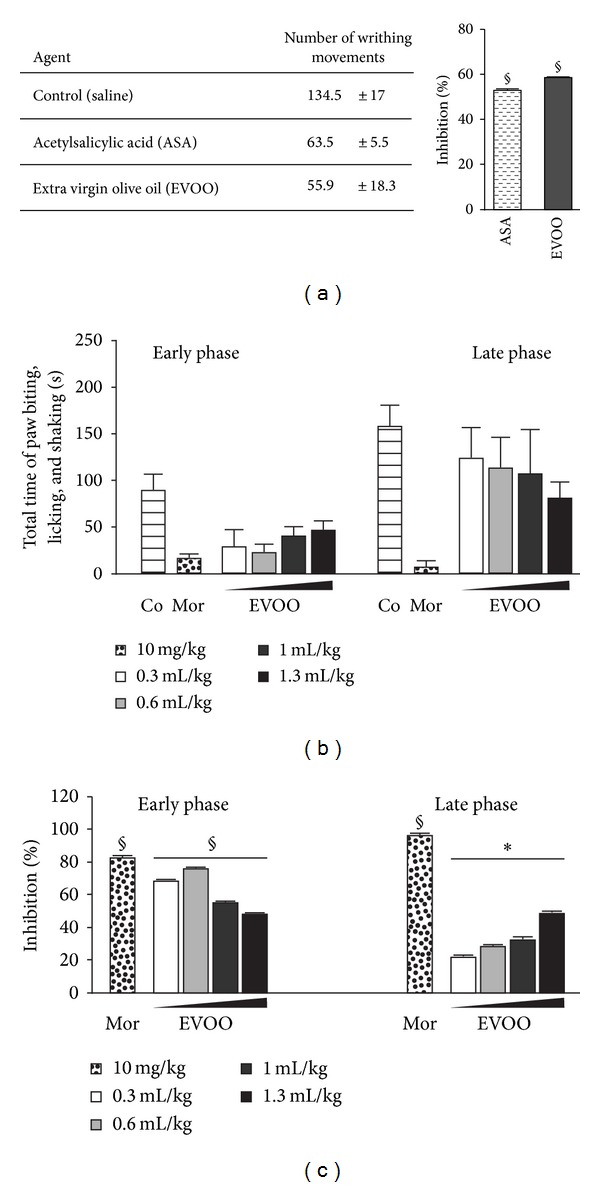
*Antinociceptive effect of extra virgin olive oil.* (a) Effect of intraperitoneal administration of acetylsalicylic acid (3.2 g/kg) and extra virgin olive oil (8 mL/kg) on acetic acid-induced Writhing. (b) Effect of intraplantar injection of morphine (10 mg/kg) and extra virgin olive oil (resp. 0.33, 0.66, 1, and 1.33 mL/kg) on formalin-Induced nociceptive behavior in mice at early and late phase. acetylsalicylic acid and morphine used as reference agents. (c) Percentage of formalin-induced nociceptive inhibition by extra virgin olive oil and reference agents as compared to saline-treated mice. Results are reported as means ± SD. *P < 0.05 and ^§^ *P* < 0.001 (two-tailed Student's *t*-test), as compared to saline-treated mice.

The antinociceptive effect of extra virgin olive oil (EVOO) was assessed via two experimental models of chemical pain stimuli namely, Writhing test and Formalin test. The writhing test showed that the abdominal constriction responses, defined in writhing movements induced by intraperitoneal injection of acetic acid, were significantly decreased in pretreated mice. The EVOO (58%) gives slightly better effect than acetylsalicylic acid (ASA) (52%) compared with the control group for, respectively, 8 mL/kg and 200 mg/kg (Figure 1(a)). ASA served as the antinociceptive standard drug in this assay. Concerning the Formalin test, the EVOO significantly reduced the nociception response for the intraplantar injection of 10% formalin inducing licking, biting, and shaking paw (Figure 1(b)). In both nontreated (naive) and pretreated mice, the formalin produces a biphasic period of intensive response which arises from 0 to 5 min after formalin injection that corresponds to the early phase, whereas the late phase was evident from 5 to 30 min with an intensive response during the last 10 min. In the early phase, EVOO at a dose of 0.6 mL/kg seems to be almost as potent as morphine at 10 mg/kg, reference drug used in this test, with 76% against 82% of licking/biting inhibition, respectively. At late phase, the effect of EVOO is less efficient than the positive control morphine but still significantly antinociceptive in a dose dependent manner and the maximal effect was observed at 1.3 mL/kg with 50% of licking/biting inhibition (Figure 1(c)).

### 3.2. Anti-Inflammatory Effect of Extra Virgin Olive Oil

**Figure 2:**
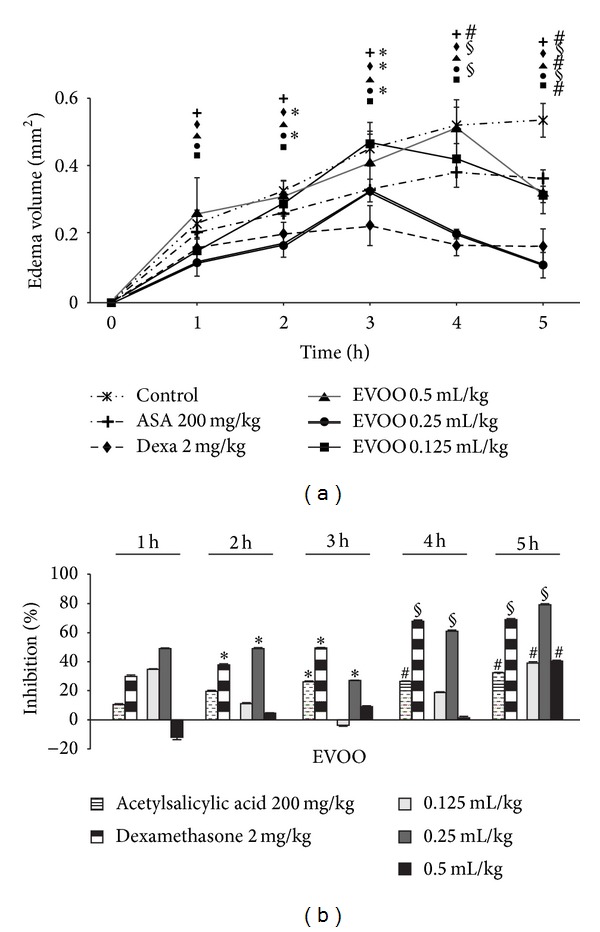
*Anti-inflammatory effect of extra virgin olive oil.* (a) Time course of the carrageenan-induced rat hind paw edema volume after intraplantar injection of acetylsalicylic acid (200 mg/kg), dexamethasone (2 mg/kg) and extra virgin olive oil (resp. 0.125, 0.25, and 0.5 mL/kg). Dexamethasone and acetylsalicylic acid were used as reference agents while saline was used as control. (b) Percentage of carrageenan-induced edema inhibition by extra virgin olive oil and reference agents as compared to saline-treated rats. Results are reported as means ± SD. *P < 0.05, ^#^ *P* < 0.01, and ^§^ *P* < 0.001 (two-tailed Student's *t*-test), as compared to saline-treated rats.

We opted for the Carrageenan-induced paw edema model in rats, to evaluate the anti-inflammatory activity, as one of the well-established acute inflammatory models *in vivo. *The intraplantar injection of 100 *μ*L of carrageenan (0.6%) into the rat's hind paw induced progressive increasing edema volume in the control group (Figure 2(a)) as inflammatory response as early as 0–5 hours. Pretreatment with EVOO at different doses of 0.125, 0.25, and 0.5 mL/kg caused a significant inhibition of the paw swelling caused by carrageenan after 4 h. EVOO 0.25 mL/kg exhibited maximum inhibition of 79% at the 5th hour of the experiment. Under the same experimental conditions, the anti-inflammatory effect of dexamethasone as well as ASA was equivalent, respectively, to 69 and 32% inhibition. At both doses 0.125 and 0.5 mL/kg the EVOO effect is less intense than the positive control though it remains significantly protective about 40%, which seemed to be more effective than ASA at the fifth hour (Figure 2(b)). We noted that at, 0.5 mL/kg, EVOO shows a proinflammatory value at (1 h) and presents an ascendant curve with an extreme value similar to the negative control group at (4 h). Nevertheless, this dose of EVOO reaches the 40% of edema inhibition at the last hour (5 h) of the experiment. This phenomenon appeared to be related to the volume of injection (100 *μ*L of oil) in the subplantar space of the hind paw, presumably relatively tight, that consequently seems to create a sort of tangible volume miming a proinflammatory effect. Hence, the inhibition percent of this dose could not refer to the plausible anti-inflammatory effect which is probably more important (Figure 2(b)). As a summary, these results are conclusive of the potent anti-inflammatory activity of EVOO in acute inflammation.

### 3.3. *In Vivo *Anticolon Cancer Effect of Extra Virgin Olive Oil

**Figure 3:**
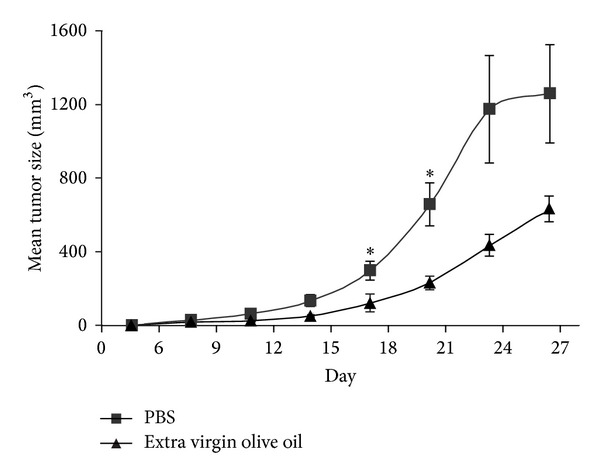
*In vivo growth inhibitory effects of extra virgin olive oil on HCT 116 human colon cancer cells. *HCT 116 cells were injected subcutaneously into athymic mice (10 mice for EVOO-treated groups and 10 mice for PBS-treated controls), and one-week later 200 *μ*L intraperitoneal injections of PBS 1X or extra virgin olive oil (1 mL/kg) were performed, as described in Materials and Methods. Tumor size was then routinely monitored by means of a common caliper. Results are reported as means ± SD. *P < 0.05 (two-tailed Student's *t*-test), as compared to PBS-treated mice.

We evaluated the anti cancer efficacy of EVOO *in vivo*. About 2 × 10^5^ HCT 116 WT cells were implanted in Athymic *nu/nu* mice subcutaneously. After that each mouse had a tumor of about 125 mm^3^, three times a week intraperitoneal injection of phosphate-buffered saline X1 (PBS) or EVOO (4 mL/kg) started. Our results show that EVOO treatment significantly inhibited the growth of colon tumors (Figure 3). Starting to third week of treatment, tumor volume in the treated group was approximately reduced by 50% as compared with control group. These data demonstrate that EVOO has a great tumor growth inhibition effect. During the whole treatment period, no significant weight changes or macroscopic signs of toxicity occurred in the treated animals suggesting that the intraperitoneal administration of EVOO was well tolerated.

## 4. Discussion

In this study, we report that the administration of EVOO have a strong analgesic and anti-inflammatory effect. The antinociception effect of EVOO was obviously noticed via two nociception models. First, the acetic acid-induced writhing as characteristic abdominal stretching and extension of hindlimbs [18] to screen for both central and peripheral analgesic activity. In fact, the presence of acetic acid provokes the release of endogenous mediators like bradykinin, substance P, prostaglandins, and some cytokines which excite chemosensitive nociceptors thus far results pain [19, 20]. Second, formalin-induced licking/biting and shaking paw in mice was conducted to distinguish analgesic from anti-inflammatory properties, where the early phase is characterized by neurogenic pain due to a direct effect on nociceptors resulting from bradekinin and substance P release, whereas the late phase is characterized by inflammatory pain response of inflammatory mediators, namely, histamine and prostaglandins liberation [14, 15, 19]. Formalin injection provokes a direct and characteristic response of nociceptors leading to stimulation of C fibers [21]. It should be mentioned that endogenous inflammatory pain-producing substances can act in a synergistic way to increase pain levels [22]. Such chemical nociceptive stimuli induced by formalin or acetic acid entail endogenous pronociceptive substances (bradekinin and prostanoids) release, which explains the characteristic behavior resulting from nociception response in mice [15, 23]. EVOO as well as ASA exhibited antinociceptive activity in abdominal constriction test (Figure 1(a)), defining blockade of peripherally and centrally mediated nociception induced by chemical stimuli. This effect on nociception process was further confirmed by the drastic effectiveness of EVOO in attenuating the early phase of formalin test and late phase with a lower but significant effect (Figures 1(b) and 1(c)). The finding that EVOO as Morphine exerted marked analgesic activity in the late phase of the formalin test suggests an effect on acute inflammation. Results from the carrageenan-induced paw edema confirm this suggestion. The carrageenan-induced inflammatory response presents two main phases: the initial phase lasts up to 2 h involving histamine, serotonin (5-HT), and bradekinin as for the second phase, it lasts from 3 to 5 h and is mediated by prostaglandins and cyclooxygenases [24, 25]. EVOO anti-inflammatory effect seems to be due to the inhibition of the cyclooxygenases enzymes release which is involved in prostaglandins synthesis [26]. The anti-inflammatory activity of EVOO shown to be potent was tested in acute and subacute inflammation models that are, respectively, formalin test (late phase) and the carrageenan-induced paw edema subsequently (Figures 1(b), 1(c), and 2). The latter test used ASA (NSAID) and dexamethasone (steroidal drug) as [21] and presents two major phases of serotonin and histamine release succeeded by accelerating paw swelling due to prostaglandins lysosomes and bradekinins [24, 25, 27]. EVOO elicits more evident effect on cyclooxygenases pathway, consequently prostaglandins inhibition [26].

The benefic, analgesic, and anti-inflammatory effect of EVOO is due to its composition, which is quit complex. Indeed EVOO can be divided into two groups namely saponifiable (about 99% of the oil) and unsaponifiable fractions. The saponifiable fraction includes fatty acids and triacylglycerols while the unsaponifiable fractions include squalene, carotenoids, chlorophylls, tocopherols, aliphatic alcohols, sterols, phenolic compounds, and volatile compounds [28]. The groups of minor compounds, especially polyphenol and triterpenes, are reported to possess active analgesic and anti-inflammatory effect. The oleuropein aglycone, a phenolic compound of EVOO, is described to have a great anti-inflammatory effect with the same experimental model as our study, meaning the carrageenan-induced [29] and also *in vitro* effect by preventing the stimulatory effect of TNF-*α* on human monocyte-like cell line [30]. Moreover, tyrosol and hydroxytyrosol, bioactive metabolites of oleuropein, were shown to have an anti-inflammatory action *in vivo* with an ovariectomy/inflammation experimental model [31]. Similarly, oleocanthal (other phenolic compound) was found to display nonsteroidal anti-inflammatory drug activity “ibuprofen like.” Ibuprofen is widely used in the therapeutic management of joint inflammatory diseases [32] and has a strong analgesic effect [33]. Recently, it has been shown that another minor compound of EVOO, the maslinic acid, a pentacyclic triterpenoid, has a broader antinociceptive effect than ibuprofen via the acetic acid-induced writhing and formalin test [34]. Erythrodiol (triterpenes alcohols) and oleanolic acid (triterpenes acid) have also shown anti-inflammatory activity reported *in vitro *studies [35].

The last part of our study was the investigation of EVOO activity in a colon cancer xenograft model assessment and showed a significant and promising inhibition of the tumor (Figure 3). It has been demonstrated that treatment of human colon adenocarcinoma cells with phenolic compounds resulted in the inhibition of all colon carcinogenetic processes such as initiation, promotion, and metastasis, triggering cell death by apoptosis [36, 37].

We should note at the end that about 97% of the main phenolic compounds of the EVOO are secoiridoids [38] and the free secoiridoids are able to cross the oil/water barrier [39] confirming the choice of the *in vivo* model for this study.

This study constitutes a starting data and model for a future investigation of the different Mediterranean extra virgin olive oil. In fact, due to importance of the environment, culture condition, and soil to the final composition of the oil, difference exists between inter/intraregion EVOO compounds. On the other context, due to the benefic effect of the oil, we can propose the EVOO as an adjuvant, vehicle, or emulsion in a treatment context and have a synergistic effect [40] or to prevent undesired effects of other used lipids [41].

## 5. Conclusion

In conclusion, the present study shows that EVOO presents a marked analgesic, anti-inflammatory, and anticancer activity confirming its beneficial properties and supports its use in traditional and self-medication.

## Abbreviations

ASA: Acetylsalicylic acid
CVD: Cardiovascular diseases
EVOO: Extra virgin olive oil
NSAID: Nonsteroidal anti-inflammatory drug
PBS: Phosphate-buffered saline.
